# Supplementary material for: The causal relationship between the human gut microbiota and pyogenic arthritis: a Mendelian randomization study
Source: Front Cell Infect Microbiol. 2024 Nov 26;14:1452480. doi: 10.3389/fcimb.2024.1452480 (PMC11629706; doi:10.3389/fcimb.2024.1452480)
Supplement: Supplementary file 1 [file DataSheet1.zip › mendelian test/ebi-a-GCST90027703.csv_forest.pdf]

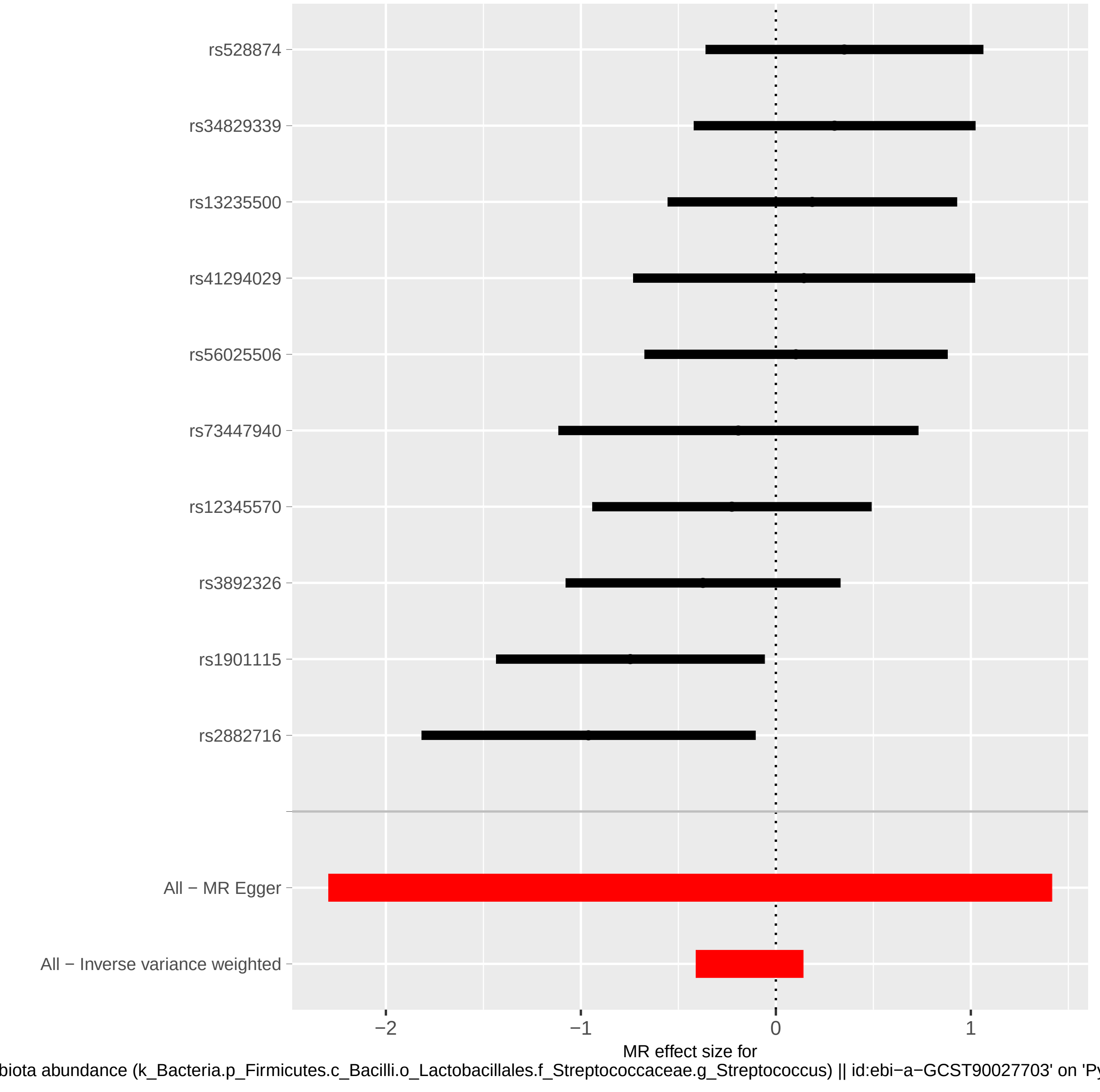

rs528874
rs34829339
rs13235500
rs41294029
rs56025506
rs73447940
rs12345570
rs3892326
rs1901115
rs2882716
All − MR Egger
All − Inverse variance weighted
−2
−1
0
1
MR effect size for
biota abundance (k_Bacteria.p_Firmicutes.c_Bacilli.o_Lactobacillales.f_Streptococcaceae.g_Streptococcus) || id:ebi−a−GCST90027703' on 'Py
